# The role of toll-like receptors in orchestrating osteogenic differentiation of mesenchymal stromal cells and osteoimmunology

**DOI:** 10.3389/fcell.2023.1277686

**Published:** 2023-10-23

**Authors:** Xiaoyang Liu, Zongke Zhou, Wei-Nan Zeng, Qin Zeng, Xingdong Zhang

**Affiliations:** ^1^ Orthopedic Research Institution, Department of Orthopedics, West China Hospital, Sichuan University, Chengdu, China; ^2^ National Engineering Research Center for Biomaterials, Sichuan University, Chengdu, China; ^3^ NMPA Key Laboratory for Quality Research and Control of Tissue Regenerative Biomaterials & Institute of Regulatory Science for Medical Devices & NMPA Research Base of Regulatory Science for Medical Devices, Sichuan University, Chengdu, China

**Keywords:** mesenchymal stromal cells, osteogenic differentiation, toll-like receptors, extracellular vesicles, biomaterials

## Abstract

Osteoimmunology is a concept involving molecular and cellular crosstalk between the skeletal and immune systems. Toll-like receptors (TLRs) are widely expressed both on mesenchymal stromal cells (MSCs), the hematopoietic cells, and immune cells in the osteogenic microenvironment for bone development or repair. TLRs can sense both exogenous pathogen-associated molecular patterns (PAMPs) derived from microorganisms, and damage-associated molecular patterns (DAMPs) derived from normal cells subjected to injury, inflammation, or cell apoptosis under physiological or pathological conditions. Emerging studies reported that TLR signaling plays an important role in bone remodeling by directly impacting MSC osteogenic differentiation or osteoimmunology. However, how to regulate TLR signaling is critical and remains to be elucidated to promote the osteogenic differentiation of MSCs and new bone formation for bone tissue repair. This review outlines distinct TLR variants on MSCs from various tissues, detailing the impact of TLR pathway activation or inhibition on MSC osteogenic differentiation. It also elucidates TLR pathways’ interplay with osteoclasts, immune cells, and extracellular vesicles (EVs) derived from MSCs. Furthermore, we explore biomaterial-based activation to guide MSCs’ osteogenic differentiation. Therefore, understanding TLRs’ role in this context has significant implications for advancing bone regeneration and repair strategies.

## 1 Introduction

Mesenchymal stromal cells (MSCs) possess robust self-renewal, immunoregulatory capabilities, and the remarkable ability to differentiate into multiple cell types. These unique characteristics render MSCs highly valuable in the field of tissue healing and regenerative medicine, especially bone regeneration and bone formation (Caplan, 2009; Najar et al., 2022). Bone is a connective tissue comprised of multiple cellular and molecular components (Wang and Thomsen, 2021). The interaction between the cellular (MSCs, osteoblasts, osteoclasts, osteocytes, and bone lining cells) and molecular components is essential in maintaining the dynamic homeostasis and regeneration of bone. The cell types contact with each other by multi-directional signal pathways to restore the native architecture of bone, in which MSCs play the key role (Xiao et al., 2022). It has been documented that bone formation primarily depends on the recruitment and differentiation of osteogenic stem/progenitor cells during bone development, reconstruction, repair, or regeneration, while MSCs have long been considered a direct source of osteogenic progenitor cells for bone formation (Almeida-Porada et al., 2020). Moreover, bone regeneration requires a cascade of biological processes including the early stage of hematoma, inflammation, soft callus and hard callus formation as well as the final bone remodeling (Schlundt et al., 2018). The fact that inflammation induced by various degrees and cell types can result in different outcomes in bone regeneration led to new lines of investigation in bone biology. And interplay between the immune system and bone regeneration has ultimately given rise to a new research field known as “osteoimmunology” (Arron and Choi, 2000). The dual-functional MSCs which modulate the immune system as well as the osteogenic differentiation may be the central role of osteoimmunology and are yet to be defined.

Toll-like receptors (TLRs) are important mediators in inflammatory pathways and are widely expressed on MSCs and immune cells. TLRs can recognize pathogen- and danger-associated molecular patterns (PAMPs, DAMPs) derived from exogenous pathogens or endogenous cellular products. Activation of TLRs, whether located on the cell surface or within the cytoplasm, can initiate signaling pathways that directly influence MSC proliferation, migration, and differentiation, and activate inflammatory molecule production through adaptor molecules and transcription factors such as nuclear factor kappa-B (NF-κB) and IFN-regulatory factor (IRFs) by MSCs or innate immune cells such as macrophages or dendritic cells. Furthermore, in response to the resulting environmental cues mediated by TLRs, such as inflammation or tissue damage, MSCs undergo activation and exhibit specific phenotypic changes. Research has demonstrated that MSCs express functional TLRs, including TLR3 and TLR4, at high levels (Zhou et al., 2021). Upon activation, these TLRs can induce phenotypic changes in MSCs and modulate their immunoregulatory functions, thereby influencing the outcome of osteogenic differentiation and bone regeneration. Moreover, activation by TLR ligands has been found to contribute to the persistent expression of pro-inflammatory cytokines by macrophages and other immune cells thus causing bone or cartilage damage that occurs in rheumatoid arthritis (RA), osteoarthritis (OA), osteoporosis, and other bone diseases. Therefore, to better understand the role of TLR in osteogenic differentiation of MSCs and osteoimmunology is of significance.

In this review, MSCs from various tissues were reviewed, and their names were indicated as they appeared in the corresponding paper. We then outlined the influence of TLR activation or inhibition on MSCs’ osteogenic differentiation potential, giving suggestions about MSC-mediated bone formation based on osteoimmunology. We also discussed TLR-related MSC-derived extracellular vesicle utilization in the treatment of tissue repair and inflammatory disease. Finally, the role of TLR pathways in biomaterial-induced bone regeneration is summarized.

## 2 TLR activation on osteogenic differentiation potential of MSCs

### 2.1 Mesenchymal stromal cells

As defined by the Mesenchymal and Tissue Stem Cell Committee of the International Society for Cellular Therapy (ISCT^®^), mesenchymal stromal cells are required to meet the following minimal criteria: 1. being plastic-adherent under standard culture conditions; 2. expressing CD105, CD73 and CD90 while lacking expression of surface molecules CD45, CD34, CD14 or CD11b, CD79 alpha or CD19 or HLA-DR; 3. possessing multi-differentiation potential into osteoblasts, adipocytes and chondroblasts under the effects of specific culture media (Dominici et al., 2006). Firstly isolated from human and mammalian bone marrow and periosteum, MSCs were officially named “mesenchymal stem cells” (Caplan, 2017). However, due to the limited capacity to differentiate into other cell types and lacking asymmetric division, MSCs are being debated whether they should be classified as “stromal” or “stem” cells. Some researchers state that the term “stem” should be reserved for cells that possess true stem cell properties, such as the ability to self-renew and differentiate into a wide range of cell types (Sipp et al., 2018; Andrukhov, 2021).

Currently, it has been established that MSCs can be derived from almost all postnatal tissues including bone marrow, adipose tissue, peripheral blood, synovial membrane, umbilical cords, dental pulp, Wharton’s jelly, dermis, placenta, amniotic fluid, and other tissues of mesoderm (Shirjang et al., 2017; Najar et al., 2022). Moreover, MSCs may be isolated from perivascular tissue and vascularized tissue since they are found to reside in blood vessel walls (Caplan, 2009). According to the extraordinary secretory, immunomodulatory, self-renewal, and multi-lineage differentiation capacity, MSCs have been widely used in tissue repairing and homeostasis such as bone, cartilage, tendon, muscle, and other mesodermal tissues, as well as immune-related disease therapies, which include acute respiratory distress syndrome (ARDS), OA, graft-versus-host disease (GVHD) and other diseases (van den Akker et al., 2013; Zhou et al., 2021). Furthermore, MSCs exhibit a tolerable safety profile due to the lack of co-stimulatory molecules, including human leukocyte antigen class-II (HLA-II), CD80, and CD86, which helps avoid activation of host T or B lymphocytes and widen the utilization of MSC *in vivo* (Bassi et al., 2011). Numerous clinical trials of MSC-related therapies have been completed in patients with degenerative, autoimmune diseases and other diseases. However, the selection and preparation of MSCs for studies and applications do not yet have a common standard.

### 2.2 Toll-like receptors

Toll-like receptors (TLRs), a kind of Pattern Recognition Receptors (PRRs), are a family of proteins playing a key role in recognizing altered patterns on microbes, viruses (Pathogen-Associated Molecular Patterns, PAMPs) or endogenous danger signals (Damage/Danger-Associated Molecular Patterns, DAMPs) thus stimulate immunity and host defense that culminate in the expression of inflammatory cytokines, chemokines or antimicrobial products (Fitzgerald and Kagan, 2020). TLRs share a similar domain organization as they are all type Ⅰ single-pass transmembrane proteins with an N-terminal leucine-rich repeat-containing ectodomain, a transmembrane domain, and a cytoplasmic C-terminal Toll-interleukin-1 receptor (IL-1R) homology (TIR) domain. Researchers have identified TLR family members in organisms ranging from corals to humans by genome browsing for proteins that share these features. 10 human TLRs (TLR1-10) and 12 mouse TLRs (TLR1-9, TLR11-13) were identified respectively, while other species vary in number in those evolutionarily conserved proteins (Kawai and Akira, 2011). Most TLRs act as homodimers, while only TLR2 paired with TLR1 or TLR6 function as heterodimers (Oliveira-Nascimento et al., 2012). TLRs that occupy the plasma membrane include TLR1, 2, and 6 which engage bacterial lipoproteins, and TLR4 and 5 which detect lipopolysaccharide (LPS) and flagellin, respectively (Lester and Li, 2014). In contrast, TLRs expressing in endosomes, lysosomes, or endoplasmic reticulum recognize nucleic acids. Specifically, TLR3 detects double-stranded RNA (dsRNA), TLR7, 8 sense single-stranded RNA (ssRNA) and TLR9 recognizes unmethylated CpG containing ssDNA (Chow et al., 2018; Greulich et al., 2019). Considered the only TLR to have the potential role that induces anti-inflammatory activity, TLR10 can homodimerize or heterodimerize with either TL1 or TLR2. The ligands that interact with TLR2 are also considered ligands of TLR10, such as Pam3Cys (Fore et al., 2020). Apart from the PAMPs, TLRs could be activated by endogenous ligands released from damaged tissues or dead cells (Yu et al., 2010).

As for signal transduction pathways, all TLRs except TLR3 use the myeloid differentiation primary response protein 88 (MyD88)-dependent pathway to mediate the production of inflammatory cytokines while TLR3 activated MyD88-independent pathway: TIR-domain containing adaptor inducing interferon (IFN)-β (TRIF or TICAM1) dependent pathway. Interestingly, TLR4 can activate both of them (Sangiorgi and Panepucci, 2016; Abdi et al., 2018; Zhang et al., 2023).

### 2.3 TLR signaling modulates proliferation and osteogenic differentiation of MSCs

**FIGURE 1:**
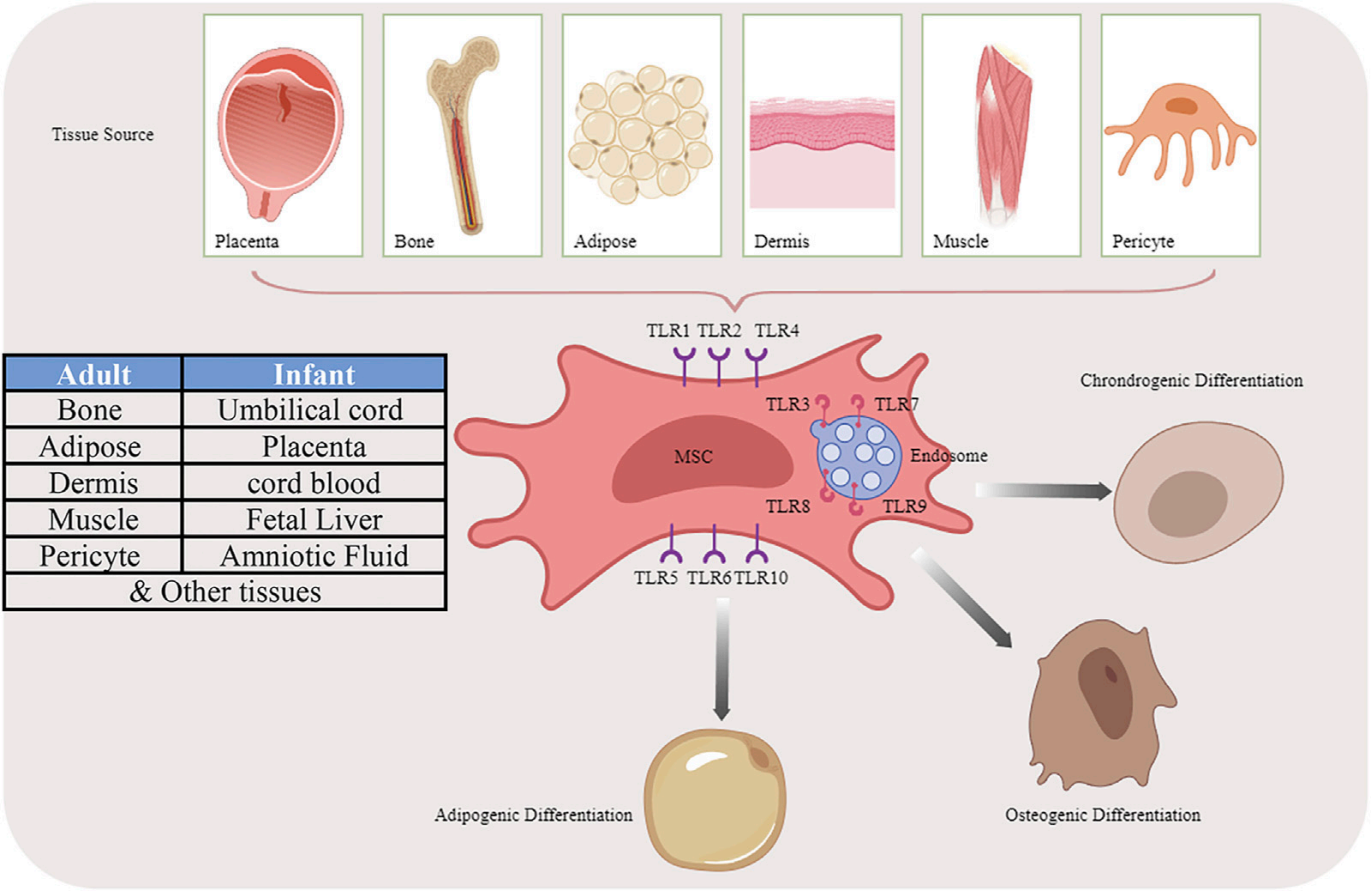
Multiple differentiation potential of MSCs originated from different sources by activating TLR pathways.

Except for innate immune cells, TLRs also express on MSCs. The exact expression pattern varies from different cell origination: bone marrow (BM), adipose tissue (AT), umbilical cord blood (UC), human placenta (hpl), Wharton’s Jelly (WJ), and other adult or fetal tissues (Guillot et al., 2008; El-Sayed et al., 2017). TLR2-4, however, is irrefutably present on the MSCs due to their responsiveness to the corresponding ligands (Jia et al., 2019). After migration to the site of tissue injury or inflammation, MSCs could be exposed to not only immune cells but also TLR ligands (Mohamad-Fauzi et al., 2023), TLRs greatly impact their functions such as proliferation, migration, angiogenesis, differentiation, and immunomodulatory capacity which lead to the MSC-based treatment in tissue regeneration or inflammation-related diseases more than ever (Figure 1).

The MSCs originated from different tissues possess the capability to undergo differentiation into osteoblasts and terminally the most abundant osteocytes upon exposure to various osteogenic signals, underlying their importance in regenerative medicine (Pipino and Pandolfi, 2015). Moreover, following fracture or bone defects, MSCs transport or migrate to the site of injury via the peripheral blood circulation, thereby augmenting the regenerative capacity of the local MSC population. Besides the bone formation effects, MSCs establish interactions between bone formation and resorption via RANKL (receptor activator of nuclear factor kappa B ligand)/RANK coupling to modulate the activity of osteoclasts (Ikebuchi et al., 2018; Schlundt et al., 2018). Considering the accompanied damage of blood vessels during bone injury, MSCs also promoted angiogenesis by secreting pro-angiogenic factors thus activating endothelial cells (Grote et al., 2013).

To date, several *in vitro* studies that isolated MSC to detect the expression of TLR profiles are listed in Table 1. Recent research also demonstrated a novel function of TLRs in modulating the osteogenic differentiation potential of MSCs, in which alkaline phosphatase (ALP), osteocalcin (OCN), collagen 1 (Col-I), osteopontin (OPN), osteonectin (ON), osterix (OSX), runt-related transcription factor 2 (RUNX-2) and mineralization assay (alizarin red staining) were used to assess osteogenesis capacity (Loebel and Burdick, 2018). Bioinformatic analyses also identified that various pathways, including the TLR signaling pathway, are associated with the osteogenic differentiation potential of BMSCs (Bone marrow-derived MSCs) (Zhang et al., 2017). The effect of activating TLR3 and TLR4 pathways in MSCs is investigated most extensively, in which most studies conducted the TLR priming protocol by administering Poly (I: C) as TLR3 ligand and LPS as TLR4 ligand. Nevertheless, the osteogenic differentiation varied according to the ligand type and concentration. In addition, age should be taken into consideration as ligation of TLR3 and TLR4 on the BMSCs, that are isolated from young people, showed higher osteogenic differentiation in comparison with the BMSCs isolated from the senior generations (Shirjang et al., 2017). MSCs exhibit different potential according to tissues they are isolated from, BMSCs and adipose tissue-derived MSCs (AMSCs) represented the optimal stem cell source for tissue engineering as they were observed to maintain the strongest differentiative abilities (Heo et al., 2016). Therefore, we categorize and discuss the osteogenic differentiation according to the MSC origin, nevertheless, an identification of the efficient source under a unified standard is needed.

**TABLE 1:** Gene and protein expression of TLRs on MSCs from different sources (+): present; (−): absent.

Source of MSCs	Gene expression	Protein expression	References
All tissue	TLR2, 3, 4, 9	TLR2, 3, 4, 9	Sangiorgi and Panepucci (2016)
UC-MSCs	TLR2, 4, 6, 9(high) Chen et al. (2013)	TLR3, 4 (high) Chen et al. (2013)	Chen et al. (2013)
	TLR2, 3, 4, 7, 8, 9 (high)	TLR4 (low) van den Berk et al. (2009)	van den Berk et al. (2009)
	1, 5, 6, 10 (low) van den Berk et al. (2009)		
h-AMSCs	TLR2, 3, 4, 7(high) TLR8 (−) Pevsner-Fischer et al. (2007)	TLR 1, 2, 3, 4, 5, 6, 9 Cho et al. (2006)	Pevsner-Fischer et al. (2007)
	TLR1, 5, 6, 10 (low) Cho et al. (2006)		Cho et al. (2006)
WJ-MSCs	TLR1, 2, 3 Raicevic et al. (2011)	TLR3(+) under inflammatory conditions Raicevic et al. (2011)	Raicevic et al. (2011)
	TLR4(+) Mei et al. (2013)	TLR4(−) Mei et al. (2013)	Tomchuck et al. (2008)
			Mei et al. (2013)
h-BMSCs	TLR1, 2, 3, 4, 5, 6, 9 Liotta et al. (2008)	TLR1, 2, 3, 4, 5, 6, 9	Cho et al. (2006)
	TLR1, 5, 6, 10 (low)	TLR3, 4 (high)	Liotta et al. (2008)
	TLR3, 4, 7, 8, 9 (high)		Shirjang et al. (2017)
	TLR1, 5, 6, 10 (low) Shirjang et al. (2017)		

#### 2.3.1 Bone marrow-derived MSCs

MSCs are conventionally exposed to inflammation in the early osteogenesis phase. In research concerning BMSCs, Waterman et al. showed that LPS of 10 ng/mL increased human-BMSCs (h-BMSC)’ osteogenic differentiation and decreased adipogenesis, while inhibition of bone, fat, cartilage formation was observed in 1 μg/mL poly (I: C) treated h-BMSCs (Waterman et al., 2010). Moreover, LPS (0.5 μg/mL) with an osteogenic inducer BMP-2 synergistically contributes to the early osteogenic differentiation of h-BMSCs (Croes et al., 2015). However, opposite results were observed in mouse-BMSCs (m-BMSCs). Migration and differentiation of both adipocytes and osteoblasts in m-BMSCs were enhanced under stimulation of 50 μg/mL Poly (I: C) and reduced when treated with 1 μg/mL LPS, the transcription factor NF-κB, which is activated through the TLR4-MyD88-dependent pathway, plays a role in inhibiting the mesodermal differentiation of MSCs (Chen et al., 2014). Specifically, 1 μg/mL LPS inhibited BMP-2 induced osteogenic differentiation of m-BMSC, and the LPS-induced inflammatory microenvironment mediated the crosstalk between TLR4/MyD88/NF-κB and BMP/Smad pathway which subsequently resulted in the negative modulation of the osteogenic ability of BMP-2 (Huang et al., 2014). Another study conducted by Chen et al. demonstrated that a lower concentration of TLR ligand, 10 ng/mL Poly (I: C), or LPS of both promoted differentiation of BMSCs to Osteoblasts. Moreover, TLR3 induced ossification of Osteoblasts into osteocytes. TLR4, on the other hand, inhibited Wnt signaling and had the opposite effect (Chen et al., 2014). This was confirmed by Mei et al., the 10 ng/mL LPS group of m-BMSCs demonstrated the lowest expression of ALP, Col I, and RUNX2, as well as the least osteogenesis. The downregulated expression levels of β-catenin and c-Myc, core proteins of the canonical Wnt signaling pathway were also observed, indicating the positive effects of the Wnt signaling pathway on the proliferation and differentiation of BMSCs (Mei et al., 2022). He and others showed contradictory results. Priming m-BMSC by 1 μg/mL LPS promoted its proliferation and osteogenic differentiation, while deletion of TLR4 eliminated the effects. Moreover, they evidenced that bone formation was upregulated by activation of the Wnt 3a and Wnt 5a pathways (He et al., 2016). A recent study conducted by Khodabandehloo and others investigated whether the TLR4 ligand LPS (exogenous agonist, 1 μg/mL) and fibronectin fragment III-1c (FnIII-1c, endogenous agonist, 1 μg/mL) influenced differentiation of h-BMSCs. TLR4 expression was upregulated after induction of h-BMSC differentiation into all three lineages. Both TLR4 ligands increased the calcium deposition and osteo-differentiation. Further studies confirmed that TLR4 silencing attenuated osteogenesis (Khodabandehloo et al., 2021). Khokhani et al. proved that compared to TLR1, 2, 4, 7, 8 activators, 0.1 μg/mL CpG or 1 μg/mL Poly (I: C) treatment in hBMSCs could facilitate the early ALP activity and osteogenic differentiation, but not that of the late phase (Khokhani et al., 2021).

Pam3Cys, a TLR2 ligand, inhibited m-BMSC differentiation into osteoblasts, chondroblasts, and adipocytes at the concentration of 10 μg/mL. However, m-BMSCs derived from the mice lacking MyD88 lacked the osteogenic differentiation ability as impaired bone formation was found in appropriate differentiation medium, suggesting that TLRs and their ligands can serve as regulators of MSC proliferation and differentiation and might affect the maintenance of MSC multipotency (Pevsner-Fischer et al., 2007). 1 μg/mL Macrophage-activating lipopeptide of 2 kDa (MALP-2), a TLR2/6 ligand showed no effect on proliferation or differentiation on h-BMSCs, but the stimulation enhanced migration of the cells (Grote et al., 2013). Zhou et al. reported the results of PGN (TLR2 agonist) treatment at a concentration of 10 μg/mL on TLR2 gene-modified canine BMSCs which were established by lentivirus. The activation of TLR2 greatly boosted the HIF-1α and BMP-2 expression and subsequently augmented the osteogenesis and angiogenesis-related gene expression of the modified BMSCs (Zhou et al., 2019). High mobility group box 1 (HMGB1), which is in the inflammatory microenvironment of bone fracture, was reported to promote h-BMSC osteo-differentiation by binding to TLR2/4 and activating the p38 MAPK signaling pathway (Lin et al., 2016).

Zhang and others discovered that knockdown of triggering receptor expressed on myeloid cells 2 (TREM-2) downregulated the expression of TLR2, 4, and 6 in h-BMSCs, resulting in the inhibition of osteogenic, chondrogenic, and adipogenic differentiation (Zhang et al., 2016). Interestingly, in a mouse calvarial critical-size defect model, activation of IL-1R/MyD88 pathway reduced m-BMSC-driven bone regeneration, and utilization of IL1R1/MyD88 inhibitors rescued the impaired healing progress and accelerated mineralization as well as bone regenerative responses. MyD88^−/−^ mice exhibit faster bone regeneration (Huh and Lee, 2013). Taken together, the TLRs and their downstream signaling components could precisely regulate the migration, differentiation, proliferation of MSCs, and crosstalk with osteogenesis-related pathways.

#### 2.3.2 Adipose-derived MSCs

Herzmann et al. reported that 0.1 μg/mL LPS enhanced proliferation and osteogenic differentiation of human-AMSCs (h-AMSCs) and the inhibition of TLR4 resulted in attenuation of differentiation, giving evidence that TLR4 may be crucial in the differentiation of AMSCs (Herzmann et al., 2017). Ligation of TLR3 (Poly I: C 1 μg/mL) and TLR4 (LPS 1 μg/mL) on h-AMSCs increased their osteogenic differentiation potential without affecting adipogenic differentiation (Lombardo et al., 2009). Bioinformatics was used to analyze mRNAs and pathways related to osteogenic differentiation in h-AMSCs. The interactions between long noncoding RNA (lncRNA)-PCAT1, microRNA(miR)-145-5p, and TLR4 were confirmed through a dual-luciferase reporter assay. Following induction of osteoblast differentiation, the expression of lncRNA-PCAT1 and TLR4 exhibited an increase, whereas the expression of miR-145-5p decreased. LncRNA-PCAT1 exerted a negative regulatory effect on miR-145-5p, leading to the promotion of TLR4 expression, and ultimately facilitated osteogenic differentiation by activating the TLR signaling pathway (Yu et al., 2018).

#### 2.3.3 h-umbilical cord MSCs

TLRs have been shown to take part in regulating the differentiation of h-umbilical cord-MSCs (UC-MSCs) in different ways (Najar et al., 2017). *In vivo* and vitro studies conducted by Guillot and others found that first-trimester fetal BMSCs exhibited more calcium deposition and expressed higher osteogenic-specific genes including OCN, ON, OPNALP, Col I, BMP2 than adult BMSCs under basal or osteogenic conditions (Guillot et al., 2008). Chen et al. demonstrated that at 24 h after stimulation, poly (I: C) inhibited UC-MSC proliferation in a dose-dependent manner while osteogenic markers were not significantly altered when administered with poly (I: C), LPS, FSL (TLR2&6 ligand), or CpG-ODN (TLR9 ligand) (Chen et al., 2013). At the concentration of 5 μg/mL, CpG enhanced the level of calcium deposition and osteogenic differentiation, while inhibiting MSC proliferation and migration of UC-MSCs (Yang et al., 2015). Zhang et al. discovered that stimulation of TLR3 inhibited the osteogenesis of UC-MSCs and TLR4 increased the process to a certain extent, although the exact concentration of TLR agonists was not mentioned (Zhang et al., 2015b). As for TLR5, flagellin did not influence the differentiation of UC-MSCs (Li et al., 2015). TLR7 agonist Imiquimod (IMQ) of 10 μg/mL enhanced the osteo-differentiation ability of UC-MSCs (Zhang et al., 2015a). Yang et al. showed that R848 (TLR8 agonist) of 5 μg/mL could increase osteocyte differentiation (Yang et al., 2017).

#### 2.3.4 MSCs from other tissue sources

Mei et al. showed that although WJ-MSCs responded to LPS by initiating upregulation of inflammatory cytokines, proliferation and differentiation were not affected (Mei et al., 2013). The effect of LPS and other TLR agonists on osteogenic differentiation of periodontal tissue-derived MSCs has also been widely studied. According to Li et al., LPS (10 μg/mL) could reduce the osteogenic differentiation and RUNX-2 expression of human periodontal ligament stem cells (h-PDLSCs) through TLR4-induced NF-κB activation, but not that of h-BMSCs (Li et al., 2014). In another study, stimulation of *P. gingivalis* LPS at 10 μg/mL induced inflammatory TLR4 pathway which impaired osteogenesis (Yu et al., 2019). *E. coli* LPS of 2 μg/mL inhibited the expression of BMP-2, OSX, and RUNX-2, leading to decreased osteogenesis in h-PDLSCs (Kim et al., 2018). Other studies evidenced the positive effects of LPS on osteogenesis. Albiero et al. reported that *E. coli* LPS (1 μg/mL) treatment in h-PDLSCs elevated the inflammatory cytokines expression of interleukin-1 beta (IL-1β), interleukin-6 (IL-6), interleukin-8 (IL-8), tumor necrosis factor alpha (TNF-α) as well as the osteogenic activities such as higher RUNX2 and ALP level (Albiero et al., 2015). However, by using *P. gingivalis* LPS of the same concentration, no effects were observed of the h-PDLSCs (Albiero et al., 2017). The proliferation of human turbinated MSCs (h-TMSCs) was significantly increased by stimulation of 10 ng/mL LPS, but the differentiation potential remained unchanged under the effects of different TLR agonists (Hwang et al., 2014).

In summary, osteogenic differentiation of MSCs can be largely enhanced or diminished under stimulation of different TLR agonists (summarized in Table 2). The differences in protocols and species employed by researchers may be the reasons for inconsistencies between different studies. However, almost all kinds of TLR agonists (TLR2, 3, 4, 7, 8, 9) showed pro-osteogenic abilities under low concentrations (less than 1 μg/mL) though high concentrations (more than 10 μg/mL, which mimics severe inflammation) of TLR agonists stimulation usually caused reduced osteogenesis. Hence, we cautiously conclude that TLR-mediated mild inflammation, to some extent, promotes osteogenesis in MSCs while severe inflammation or intense stimulation of TLR pathways impairs bone formation of MSCs. Table 3.

**TABLE 2:** Osteogenic differentiation stimulated by individual TLR ligands of certain concentration on MSCs.

Source of MSCs	TLR stimulation	Osteogenic differentiation	References
h-BMSCs	TLR4: 10 ng/mL LPS	Increase	Waterman et al. (2010)
	TLR3: 1 μg/mL poly(I:C)	Decrease	Grote et al. (2013)
	TLR2/6: 1 μg/mL MALP-2	NS	Khodabandehloo et al. (2021)
	TLR4: 1 μg/mL LPS	Increase	Khokhani et al. (2021)
	TLR4: 1 μg/mL FnIII-1c	Increase	
	TLR3: 1 μg/mL Poly (I:C)	Increase	
	TLR9: 0.1 μg/mL CpG ODN	Increase	
m-BMSCs	TLR2: 10 μg/mL Pam3Cys	Decrease	Pevsner-Fischer et al. (2007)
	TLR3: 50 μg/mL Poly (I:C)	Increase	Chen et al. (2014b), Huang et al. (2014)
	TLR4: 1 μg/mL LPS	Decrease	He et al. (2016)
	TLR4: 1 μg/mL LPS	Increase by Wnt5a and Wnt 3a	Mei et al. (2022)
	TLR4: 10 ng/mL LPS	Decrease	Chen et al. (2014a)
	TLR3: 10 ng/mL Poly (I:C)	Increase	
	TLR4: 10 ng/mL LPS	Increase	
Canine-BMSCs	TLR2: 5 μg/mL PGN	Increase	Zhou et al. (2019)
h-AMSCs	TLR4: 0.1 μg/mL LPS	Increase	Herzmann et al. (2017)
	TLR4: 1 μg/mL LPS	Increase	Lombardo et al. (2009)
	TLR3:1 μg/mL poly(I:C)		
UC-MSCs	TLR2,3,4,6,9	NS, TLR3 decrease proliferation	Chen et al. (2013)
	TLR9: 5 μg/mL CpG	Increase	Yang et al. (2015)
	TLR5: flagellin	NS	Li et al. (2015)
	TLR7: 10 μg/mL IMQ	Increase	Zhang et al. (2015a)
	TLR8: 5 μg/mL R848	Increase	Yang et al. (2017)
WJ-MSCs	TLR4: LPS	NS	Mei et al. (2013)
h-PDLSCs	TLR4: LPS 10 μg/mL	Decrease by TLR4-NF-κB pathway	Li et al. (2014)
	TLR4: *E. coli* LPS (2 μg/mL)	Increase	Kim et al. (2018)
	TLR4: *E. coli* LPS (1 μg/mL)	Increase	Albiero et al. (2015)
	TLR4: *P. gingivalis* LPS (1 ug/mL)	NS	Albiero et al. (2017)

NS: not significant.

**TABLE 3:** MSC extracellular vesicles function by up-, down-regulating TLR signaling or regulated by TLR pathway.

	miR	Source of MSCs	TLR priming	Disease model	Functional outcomes	Related pathways	References
**MSC-derived EVs regulate TLR signaling**	miR-21-5p	mBMSC-EVs	LPS	LPS-induced inflammation	Upregulate IL-6, IL-10, HMGB1. Suppression of miR-21-5p resulted in inhibition of TLR4 and over-expression of Wnt5a	Wnt-5a	Fafian-Labora et al. (2017)
	miR-301a	mA/BMSC-EVs	LPS/poly(I:C)	LPS-induced inflammation	Upregulate IL6, IL-8, IDO1, COX-2, PGE2, IFN-β. Upregulate TLR4 signaling	TLR4	Hsu et al. (2020)
							Huang et al. (2013)
	Let-7b	UCMSC-EVs	LPS	—	Reduce macrophage inflammatory factor level. Induce M2 phenotype	TLR4-p65/AKT-STAT	Chiossone et al. (2016)
	miR-150-5p	hBMSC-EVs	Flagellin	Ischemia-induced MCAO	Exert a protective role against brain I/R injury by inhibiting TLR5	TLR5	Li et al. (2022)
	miR-17-5p	hBMSC-EVs	—	IDD	Enhance the proliferation of HNPCs and ECM synthesis. Alleviate IDD.	TLR4; PI3K/AKT	Zhou et al. (2022)
	Not mentioned	UCMSC-EVs	—	Cyclophosphamide-induced IC	Alleviated suprapubic mechanical allodynia and frequent micturition. Inhibit NLRP3 inflammasomes	TLR4/NF-κB	Zhang et al. (2022)
	miR-182-5p	hBMSC-EVs	—	H_2_O_2_-stimulated NMVM; MI	Promote cardiac function in MI mice, inhibit the inflammatory response, and inactivate the TLR4 pathway	TLR4/NF-κB	Sun et al. (2022)
	miR-93-5p						Liu et al. (2018a)
	miR-125b-5p	UCMSC-EVs	—	Ischemia-induced MCAO	Downregulate TNF-α, IL-1β and IL-6 of astrocyte. Suppress inflammation thus maintaining BBB integrity	TLR4/NF-κB	Qiu et al. (2022)
	miR-21a-5p	mBMSC-EVs	LPS	LPS-induced sepsis	Suppress inflammation by targeting TLR4 and PDCD4	TLR4	Niu et al. (2023)
	miR-342-5p	hAMSC-EVs	—	LPS-induced AKI	negatively regulated TLR9 to induce autophagy, thereby attenuating inflammation induced by LPS.	TLR9	Liu et al. (2023)
	Not mentioned	hplMAC-EVs	LPS	LPS-induced inflammation	Downregulate TNF-α, IL-1β and IL-6 of RAW 264.7 cells, inhibit M1 polarization	TLR4/NF-κB MAPK/PI3K	Hu et al. (2022)
	miR-214-3p	hTMSC-EVs	IMQ	IMQ-induced inflammation	Reduce inflammatory cytokines, mast cell, and CD-14 cell number under inflammatory condition	TLR7	Cho et al. (2022)
	miR-424-5p						
	Not mentioned	UCMSC-EVs	—	LPS-induced CCI	Restrain TLR2 signaling activation in the spinal microglia. Reduce CCI via intrathecal injection	TLR2/MyD88/NF-κB	Gao et al. (2023)
	miR-181c	UCMSC-EVs	LPS	Burn-induced inflammation	Inhibited the TLR4 signaling pathway and alleviated inflammation in burn-injured rats	TLR4	Li et al. (2016b)
**EVs from TLR ligands primed MSCs**	miR-1180, miR-183	hUCMSC-EVs	LPS	—	Upregulated following LPS treatment	Not identified	Chiossone et al. (2016)
	miR-550b, miR-133a						
	Let-7b	UCMSC-EVs	LPS	Inflammation and diabetic wound healing	Upregulate expression of anti-inflammatory cytokines and promote M2 macrophage activation, enhance diabetic cutaneous wound healing	TLR4/NF-κB/STAT3/AKT	Ti et al. (2015)
	Not mentioned	hBMSC-EVs	Poly(I:C)	ALI by intratracheal instillation of *E. coli*	Enhance monocyte phagocytosis of bacteria, and reduce influx of inflammatory cells, cytokines, and proteins	TLR3	Monsel et al. (2015)
	miR-219a-2-3	mBMSCs	LPS	LPS-induced neuro-inflammation	Downregulate IL1β, IL-6, TNF-α. Inhibit astrocyte and microglial activation as well as amyloid deposition and demyelination	TLR4	Markoutsa et al. (2022)
	miR-10527						
	miR-329-5p						
	Not mentioned	hBMSC-EVs	Poly(I:C)	*Staphylococcus aureus* infection	Increase protein expressions of the innate and adaptive immune system	TLR3	Pierce and Kurata (2021)

## 3 TLR activation on MSC immune regulation, and the immune system’s role in regulating MSC osteogenesis

As one of the most promising bone tissue engineering strategies, priming MSCs with TLR agonists has shown extraordinary capacity to promote bone healing and regeneration. However, due to the low survival and engraft rates of the transplanted MSCs despite successful outcomes observed *in vivo* (Brennan et al., 2014), the initial premise that MSC improves damaged tissue regeneration through osteogenic differentiation has been weakened, replaced by the paracrine mechanism which largely stimulates recruitment of host cells and ultimately forms new bone tissue (Najar et al., 2022; Shen et al., 2022). The underlying mechanisms are yet to be elucidated. Nevertheless, research results demonstrated that the MSCs and their paracrine productions (cytokines, chemokines, extracellular vesicles) regulate innate and adaptive immune cells, modulate inflammatory response, and accelerate angiogenesis, which synergistically enhances bone formation and osteointegration. The reports of MSCs modulating immune response and immune response ameliorating MSC-initiated bone regeneration coincide with the concept “osteoimmunology” proposed by Takayanagi et al. (Arron and Choi, 2000), in their study the regulation of T lymphocytes on osteoclast activation was discovered, emphasizing the dynamic relationship between bone and immune system.

### 3.1 Osteoimmunology: the immune system regulates the bone-forming/adsorbing progress

To date, regulation of osteoimmunology has become the central role of recovering functions in defective and diseased bone (Lee et al., 2022). Apart from MSCs which have the potential to become osteoblasts, bone marrow, the semi-solid tissue enclosed in bone, encompasses hematopoietic stem cells (HSCs) and immune cells. Monocytes from hematopoietic origin partly differentiate into osteoclasts, while other groups from HSCs have the crucial function of producing different components of blood (red blood cell et al.) or maintaining the hemostasis of immune microenvironment by differentiating into immune cells such as neutrophils, mast cells, monocytes, T cells, and B cells (Dzierzak and Bigas, 2018). In addition to the immune cells maturation which takes place within the bone marrow, bone formation and immune system also share cytokines and other signal molecules due to their anatomical proximity (Hosseinpour et al., 2022).

Successful bone regeneration requires continuous activation of different types of immune cells and subsequent cytokine patterns. On the other hand, abnormal immune cell function will lead to an imbalance between osteoclasts and osteoblasts, ultimately leading to osteolysis, osteoporosis, osteoarthritis, rheumatoid arthritis, and other diseases. First discovered as early as 1972, in which IL-1 was found to be secreted by leukocytes, supporting activation of osteoclasts (Horton et al., 1972), crosstalk between bone-forming/adsorbing and immune system assists sequential activation of different components in the stages of bone regeneration or bone remodeling. Besides IL-1, one of the primary findings of cytokines, chemokines, and transcription factors involved in osteoimmunology is RANKL/RANK/osteoprotegerin (OPG) signal axis, which mediates osteoblast-controlled osteoclastogenesis and bone degradation (Witkowska-Sedek et al., 2018), has also been found to be expressed by activated T cells (RANKL) under inflammatory conditions thus promoting osteoclastogenesis and subsequent bone loss in various inflammatory and autoimmune conditions (Arron and Choi, 2000). While B cells communicate with osteoclasts and osteoblasts through OPG (Li et al., 2007; Frase et al., 2023). IL-6 was found to be one of the most important mediators of osteoclast formation and function, and over-secretion led to impaired osteogenesis in m-BMSCs (Li et al., 2016c). Nevertheless, IL-6 was reported to participate in TLR-induced osteogenesis of m-AMSCs (Huh and Lee, 2013). SH2-containing inositol phosphatase-1 (SHIP1), an immunomodulatory molecule, activated osteogenesis as a downstream of TLR2 signaling (Muthukuru and Darveau, 2014), while disruption of the modulatory molecules, such as SHP1 and NFATc1, results in mitigation of bone volume by osteoblast deactivation (Lee et al., 2022). A variety of key cytokines released by activated immune cells can activate the function of osteoclasts [OSM (Oncostatin M), IL-6, TNF-α (Fujii et al., 2012)] or exhibit inhibitory effects(IFN- γ, IL-4, IL-10, granulocyte-macrophage colony-stimulating factor (GM-CSF), IL-12 and IL-18) (Freire et al., 2016; Mae et al., 2021). Anti-inflammatory factors, such as IL-10, can also promote osteogenesis.

There are two main aspects of immune cells’ impact on bone regeneration: the anabolic function of acute inflammation during bone regeneration and the catabolic function of chronic inflammation during bone resorption (Wu et al., 2014; Kouroupis et al., 2019). Specifically, acute inflammation leads to the production of chemokines or cytokines, which accelerate bone formation in early fracture healing by stimulating the proliferation and osteogenic differentiation of MSCs and progenitor cells. Conversely, chronic inflammation increases cytokines secretion to activate osteoclastogenesis and inhibits the production of factors that stimulate osteoblasts to form new bone, thereby inhibiting bone coupling during inflammatory diseases such as periodontitis and promoting the formation of osteolytic lesions (Karlis et al., 2020; Yang et al., 2021). Together, the immune and skeletal systems crosstalk mutually, suggesting the importance of osteoimmunology not only in bone regeneration but this concept also could be extended to other tissue engineering and diseases.

### 3.2 TLR priming enhances MSCs’ immunoregulatory capacity on various immune cells

MSCs release a wide range of paracrine factors into their conditioned media (MSC-CM) *in vitro*. Intriguingly, the administration of MSC-CM *in vivo* has been shown to promote tissue healing in various tissues, including bone. This compelling evidence indicates that the secretome of MSCs can trigger the cascade of bone tissue regeneration (Ando et al., 2014). Moreover, MSCs possess immunosuppressive properties and can dampen immune responses. They can inhibit the proliferation and activation of immune cells, suppress the release of pro-inflammatory cytokines, and induce the production of anti-inflammatory factors (Garg et al., 2022). By doing so, MSCs help to create an immunoregulatory microenvironment that is favorable for bone healing and regeneration. MSCs regulate innate immunity in various ways, in which TLR signaling may be of great importance. They are capable of promoting macrophage polarization into the anti-inflammatory and pro-healing phenotype or modulating the maturation of neutrophils, monocytes, macrophages, and nature killer cells (Kota et al., 2014; Jiang et al., 2016). Zhao et al. demonstrated that poly (I: C) enhanced the anti-inflammatory effect of UC-MSCs on macrophages by up-regulating cyclooxygenase-2 (COX-2), indoleamine 2,3-dioxygenase (IDO) and IL-6 (Zhao et al., 2014). Munir et al. reported that LPS-primed h-AMSCs activated neutrophils and promoted NET (neutrophil extracellular trap) formation, which subsequently stimulated macrophage to secrete Transforming growth factor-β (TGF-β) and the continuous activation ultimately led to accelerated wound healing of mice (Munir et al., 2020). Conditioned media (CM) from h-AMSCs activated by TLR5 ligand flagellin (F-CM) were compared to CM from untreated MSCs. F-CM promoted a higher proportion of anti-inflammatory M2 macrophages, inhibited inflammatory cell recruitment, decreased inflammatory factor levels, and finally alleviated acute lung injury (ALI) (Li et al., 2020). Lu et al. stated that TLR4-treated hBMSCs exacerbate the inhibition of natural killer (NK) cell proliferation and function(Lu et al., 2015). On the other hand, MSCs also modulate the adaptive immune system. They proficiently suppress the proliferation of T cells, as observed in animal models of GVHD (Sangiorgi and Panepucci, 2016).

Additionally, MSCs can directly interact with immune cells and promote their transition to anti-inflammatory or regulatory phenotypes. For example, Pre-conditioning of m-BMSCs with poly (I: C) exhibited a notable reduction in the proliferation of CD3^+^ T cells and diminished the differentiation and activation of proinflammatory lymphocytes, specifically Th1 and Th17 (Maria Vega-Letter et al., 2016). The pre-activation of h-AMSCs with Poly:(I: C), enhanced their ability to inhibit the proliferation of T lymphocytes as well (Mancheno-Corvo et al., 2015). Moreover, MSCs can induce the differentiation of T cells into regulatory T cells (Tregs), Rashedi et al. revealed that the generation of Tregs in cocultures of human CD4^+^ lymphocytes and h-BMSCs was enhanced through the activation of either TLR3 or TLR4 in MSCs, and this enhancement was eliminated by gene silencing of TLR3 and TLR4. The amplified induction of Tregs by TLR-activated MSCs was correlated with an upregulation in the gene expression of the Notch ligand, Delta-like 1, while the inhibition of Notch signaling abolished the increased levels of Tregs in the MSC cocultures (Rashedi et al., 2017). In addition, MSCs treated with poly (I: C) exhibited a detrimental impact on the expansion of T-helper type 1/17 (Th1/17) cells while enhancing the suppressive effects of Tregs both *in vitro* and *in vivo*. The production of PGE-2 by UC-MSCs in response to poly (I: C) was found to increase the secretion of IL-10 and facilitate the differentiation of Tregs, a process that could be reversed by inhibiting Notch-1 (Qiu et al., 2017). Following a 48-h exposure to LPS, m-BMSCs acquired an anti-inflammatory phenotype, characterized by a substantial enhancement in their immunosuppressive capacity on T cell proliferation *in vitro*. Moreover, this time-dependent LPS stimulation led to improved therapeutic effects and higher levels of regulatory T cells (Tregs) when compared to unstimulated MSCs (Kurte et al., 2020). MSCs possess the ability to activate B cells, as evidenced by the increased expression of B cell activating factor (BAFF) in the presence of LPS. This observation provided validation for the involvement of signaling pathways such as NF-κB, p38 MAPK, and JNK in the TLR4-mediated induction of BAFF (Yan et al., 2014). IL-17, a pro-inflammatory cytokine, was markedly diminished in the skin-draining lymph nodes of mice that received TLR3-primed WJ-MSCs (Park et al., 2019).

### 3.3 Immune system modulates MSC-mediated bone formation

Recent studies have revealed that osteogenesis could be enhanced by activating a proper immune response in the microenvironment. Of the several main immune cells, due to their high plasticity and multi-function, macrophages hold crucial importance as an innate immune cell population involved in the regulation of MSC-based bone regeneration. Macrophages have been extensively characterized into distinct phenotypes based on their receptor expression, cytokine production, effector function, and chemokine repertoires. The two broad categories are M1 and M2 macrophages. M2 macrophages can further be classified into sub-populations, including M2a, M2b, M2c, M2d, and M2f, which are determined by the specific differentiation biomolecules and expressed markers associated with their differentiation process (Murray and Wynn, 2011).

Upon activation, the classically activated M1 macrophage subset demonstrates elevated production of pro-inflammatory mediators such as TNF-α, IL-6, and IL-1β. Conversely, alternatively activated M2 macrophages perform functions related to extracellular matrix (ECM) synthesis and remodeling. They release anti-inflammatory, angiogenic, and growth factors, including IL-10 and TGF-β (Nadine et al., 2022). Early studies demonstrated that sustained activation of M1-type macrophages leads to chronic inflammation and fibrosis, which can have a detrimental impact on the outcome of bone regeneration (Wang et al., 2022). In contrast, the M2 phenotype can reduce inflammation and promote tissue repair. The polarization of macrophages plays a critical role in the regulation of inflammation and serves as a significant target for MSC-based bone regeneration (Hanson et al., 2014). M2 macrophages upregulated IL-4, IL-10 and released higher amounts of BMP-2, vascular endothelial growth factors (VEGF), and TGF-β, thus enhanced osteogenesis and biomineralization of MSCs (Zhu et al., 2021; Wang et al., 2022). Contrary to previous assumptions, recent research has indicated that the presence of M1 macrophages can promote osteogenesis, while an excessive shift towards the M2 phenotype may result in fibrous tissue healing (Mokarram and Bellamkonda, 2014). Cu-incorporated biomaterials exhibited a positive impact on osteointegration, characterized by enhanced expression levels of M1 surface marker CD11c, growth factor BMP-6, as well as osteogenic markers such as OCN and Runx-2 at the interface between the biomaterial and bone tissue in a rat model, suggesting that activating M1 phenotype plays a key role in MSC recruitment and osteogenesis (Huang et al., 2019). Guihard et al. provided evidence that inflammatory M1 macrophages secrete OSM, which promotes osteogenesis of MSCs in an *in vitro* setting (Guihard et al., 2012). Considering recent studies, it is hypothesized that both M1 and M2 macrophages play crucial roles in the bone healing process. Therefore, precise regulation of the timing of M1/M2 transition rather than the specific phenotype is deemed essential for optimal outcomes in bone regeneration. An ideal paradigm might be activating the inflammatory phase at the early stage of repair, when the macrophages are mainly M1 phenotype, recruiting MSCs and restraining the inflammation to a mild manner. Subsequently, a quick switch to M2 results in an osteogenesis-enhancing cytokine release pattern, helping new bone formation.

**FIGURE 2:**
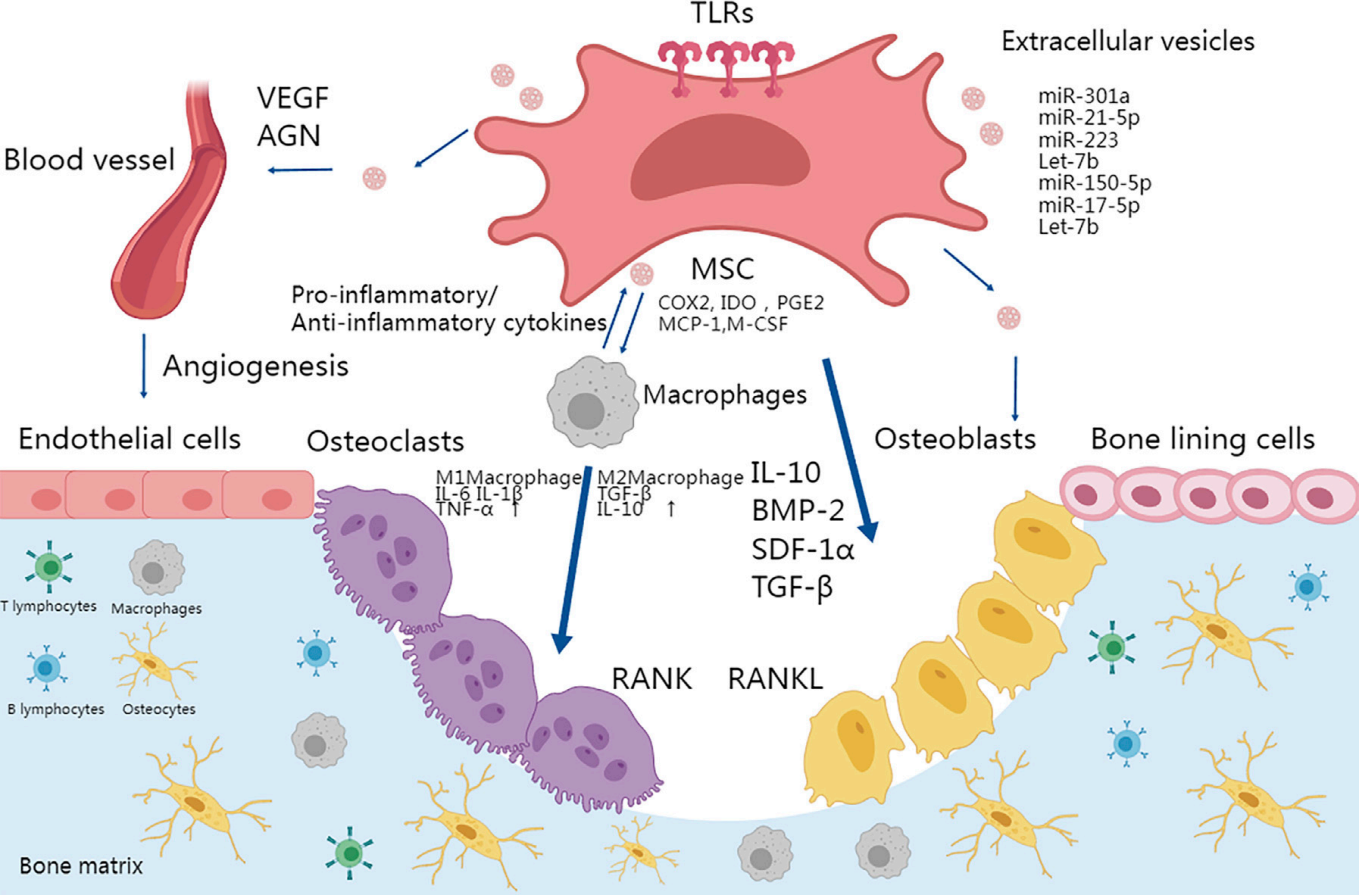
TLR activation in MSCs plays an important role in osteoimmunology. Osteogenesis could be enhanced by activating a proper immune response and MSC differentiation in the microenvironment. MSCs can be activated by TLRs and thus differentiate into osteoblasts and terminally into osteocytes. The secretion of IL-10, TGFβ, and BMPs also promotes the migration and differentiation of osteoblastic progenitor cells. TLR-primed MSCs can regulate migration, proliferation of immune cells including T cells and B cells, and polarization of macrophages into the pro-inflammatory M1 phenotype or anti-inflammatory M2 phenotype. M1 and M2 macrophages secrete IL-6, IL-1β, TNF-α, TGF-β, and IL-10 respectively to differentially affect the activity of osteoclasts. Moreover, TLR stimulation promoted MSC-mediated VEGF and other factors to enhance angiogenesis. Finally, MSC-derived vesicles participate in bone formation by activating osteogenesis directly or via immune cells.

Other immune cells also contribute to MSC-intermediated bone formation. Depletion of DCs (dendritic cells) promoted MSC-induced bone formation (Zhao et al., 2021). Infusion of CD4^+^ T cells in mice blocked ectopic bone formation through secretion of TNF-α and IFN-γ, which inhibited MSC differentiation and induced MSC apoptosis, while infusion of CD4^+^ CD25^+^ Treg abolished TNFα and IFN-γ production and improved MSC-mediated bone regeneration (Liu et al., 2011). The secretion of IL-17 by T-lymphocytes represents another crucial aspect during osteogenic differentiation. T-lymphocytes that release IL-17 actively promote the proliferation and osteoblastic differentiation of m-BMSCs (Ono et al., 2016). The dynamic microenvironment of the bone matrix is summarized in Figure 2.

## 4 MSC-derived extracellular vesicles regulate or are regulated by TLR signaling

Although MSCs are considered immune-privileged cells as previously mentioned due to the low level of co-stimulatory molecule expression (Bassi et al., 2011), safety concerns associated with live cell therapy are unavoidable and somehow limit the MSC application (Jafarinia et al., 2020). For example, in the *in vivo* environment, the hereditary factors and self-renewal capacity of MSCs cannot be fully controlled, which carries the inherent risk of potential tumorigenicity (Jeong et al., 2011). The observation that MSC-conditioned medium (CM) or factors secreted by MSCs alone can induce beneficial effects comparable to those of MSCs highlights the significance of MSC-secreted paracrine factors in mediating regenerative effects (Wang and Thomsen, 2021). For example, CM from hBMSCs which were pre-treated with pro-inflammatory cytokines could improve the proliferation and migration of MG63 cells. The expression levels of osteogenic differentiation markers ALP, Col-I, OCN, and OPN were also increased (Li et al., 2016). In particular, the paracrine mechanisms are partially mediated by extracellular vesicles (EV), which are small lipid bilayer membrane-bound vesicles secreted from the cell and play a crucial role in intercellular communication (Pierce and Kurata, 2021). EVs can be classified by their diameter into 3 subgroups: exosomes of endosome origin (less than 150 nm in diameter) (Hessvik and Llorente, 2018), microvesicles, and apoptotic bodies which are released directly from the plasma membrane of living and dying cells, respectively (more than 200 nm in diameter) (Kou et al., 2022; Ma et al., 2023).

EVs contain a diverse cargo of bioactive molecules, including proteins, lipids, nucleic acids (such as miRs), and growth factors that can be delivered to target cells (Pathan et al., 2019). MiRs, in special, are small non-coding RNA sequences that regulate gene expression by pairing with target messenger RNAs (mRNAs) and inhibiting their translation into proteins. They have garnered significant attention as key regulators present in EVs (Abdi et al., 2018). For instance, MSCs package their damaged mitochondria into vesicles and release them for engulfment by macrophages. Simultaneously, MSCs also release miR-containing EVs that inhibit TLR signaling. These miRs render the macrophages insensitive to the mitochondria they have engulfed, thereby preventing macrophage activation by suppressing TLR signaling (Phinney et al., 2015). MSC-derived EVs provide great application prospects depending on their paracrine action which is capable of replacing the MSCs to achieve the therapeutic effects. Moreover, EVs offer advantages over MSCs due to their low risk of tumorigenicity as they cannot self-replicate, higher safety as non-cell therapy, and low immunogenicity to allow allogeneic transplantation. Additionally, MSC-EVs can be easily isolated from MSC culture supernatants and can be stored for future use, allowing for off-the-shelf availability. MSC-EVs also hold great promise as nanotherapeutic agents with the potential to pass through membranes such as the blood-brain barrier (BBB) (Tsiapalis and O'Driscoll, 2020). Similar to TLR stimulation’s effects on immune regulatory capacity, differentiation, and regenerative potential, the effects of TLR signaling activation can also exert their influence on MSC-derived extracellular vesicles.

### 4.1 MSC-derived EVs regulate TLR signaling

A limited number of TLR-susceptible miRNAs identified in MSCs are involved in the positive regulation of TLR signaling. Fafian et al. reported that stimulation with LPS resulted in an upregulation of miR-21-5p, and inhibition of miR-21-5p led to a decrease in the expression of TLR4 as well as downstream cytokines (IL-6, IL-1β) by m-BMSCs. Moreover, suppression of miR-21-5p resulted in an upregulation of Wnt5a transcription, suggesting that the enhancing effect of miR-21-5p on TLR signaling is mediated by the negative regulation of Wnt (Fafian-Labora et al., 2017). According to Li et al., mAMSC-EVs transfected with miR-301a inhibitor induced M2 macrophage polarization, evidenced by strong elevation of Arg-1, CD163, CD206, and IL-10. Inhibition of miR-301a in mAMSC-EVs also suppressed TLR4 expression in macrophages, suggesting that miR-301a regulates immunomodulatory functions of mAMSC-EVs via the TLR4 pathway (Hsu et al., 2020). Similarly, the overexpression of miR-301a led to an elevation in the expression or secretion of IL-6, IL-8, COX-2, PGE2, IFN-β, and significantly induced the expression of IDO by UC-MSCs (Huang et al., 2013). On the other hand, the majority of miRNAs have a reducing effect on TLR signaling activation, implying their role in downregulating TLR-mediated immune responses in MSCs. In the middle cerebral artery occlusion (MCAO) model, miR-150-5p was found to be reduced while the TLR5 level was augmented. Treatment of hBMSC-EVs ameliorated neurological function, and pathological changes, and reduced inflammatory factors. Enrichment of miR-150-5p in the EVs exerted a protective role against brain ischemia/reperfusion (I/R) injury by inhibiting TLR5. The upregulation of TLR5 counteracts the protective effect of elevated miR-150-5p in brain I/R injury (Li et al., 2022). To mimic the hypoxic microenvironment *in vivo*, Zhou et al. isolated hBMSC-EVs under hypoxic status (H-sEVs). In intervertebral disc degeneration (IDD) model, by inhibiting the TLR4 pathway and activating the PI3K/AKT signaling pathway, H-sEVs miR-17-5p enhanced the proliferation of human nucleus pulposus cells (HNPCs) and promoted ECM synthesis, contributing to the alleviation of IDD by facilitating the proliferation and synthesis of the HNPCs’ matrix (Zhou et al., 2022). UCMSC-EVs alleviated suprapubic mechanical allodynia and frequent micturition in interstitial cystitis (IC) rats by inhibiting the TLR4/NF-κB pathway (Zhang et al., 2022). M-BMSCs transfected with miR-182-5p overexpressed it in their EVs. The expression of TLR4 in both MSCs and neonatal mouse ventricular myocytes (NMVM) was reduced by miR-182-5p. Treatment of MSC-EVs enhanced cell viability and suppressed the expression of inflammatory cytokines in NMVM posed with H_2_O_2_. Additionally, they promoted cardiac function in mice with myocardial infarction (MI), inhibited the inflammatory response, and inactivated the TLR4/NF-κB signaling pathway (Sun et al., 2022). Qiu and others used UCMSC-EVs to treat intracerebral hemorrhage following BBB disruption induced by thrombolysis of ischemic stroke with tissue plasminogen activator (tPA). In the MCAO model, the homing of EVs to the ischemic brain was enhanced by tPA. The administration of accumulated EVs demonstrated a reduction in tPA-induced disruption of BBB integrity and alleviation of hemorrhage by suppressing astrocyte activation and inflammation. The underlying mechanism involved the delivery of miR-125b-5p by MSC-EVs, which played a crucial role in maintaining BBB integrity by targeting Toll-like receptor 4 (TLR4) and inhibiting NF-κB signaling in astrocytes (Qiu et al., 2022). MiR-21a-5p, which was reported to activate the TLR pathway, was also found to suppress inflammation by targeting TLR4 and programmed cell death 4 (PDCD4). Furthermore, the therapeutic efficacy of EVs in sepsis was partially diminished when transfected with miR-21a-5p inhibitors (Niu et al., 2023). Liu et al. reported that miR-342-5p secreted by h-AMSC-derived EVs negatively regulated TLR9 to induce autophagy, thereby attenuating inflammation induced by LPS and improving acute kidney injury (AKI) in a mouse model (Liu et al., 2023). hplMSC-EVs could ameliorate LPS-induced inflammation in RAW264.7 cells by regulating TLR4-mediated NF-κB/MAPK and PI3K signaling pathways (Hu et al., 2022). Human tonsil-derived MSC (hTMSC)-EVs were discovered to have regulatory effects in mast cells under TLR7 stimulation. hTMSC-EVs containing miRs reduced inflammatory cytokines, mast cell, and CD-14 cell numbers in IMQ-treated cells and mice (Cho et al., 2022). Gao et al. discovered that UCMSC-derived EVs restrained TLR2/MyD88/NF-κB signaling activation in the spinal microglia, and intrathecal injection reduced chronic constriction injury (CCI)-induced mechanical allodynia, though the exact molecule involved was not studied (Gao et al., 2023). In another severe burn model, the injury significantly amplified the inflammatory response in rats or macrophages exposed to LPS, leading to increased levels of TNF-α and IL-1β, while reducing the levels of IL-10. The administration of UCMSC-EVs successfully reversed this reaction. miR-181c plays a pivotal role in regulating inflammation. The overexpression of miR-181c in UCMSC-EVs more effectively inhibited the TLR4 signaling pathway and alleviated inflammation in burn-injured rats (Li et al., 2016b).

### 4.2 EVs are derived from TLR ligands-primed MSCs

LPS primed-MSCs have shown paracrine effects, including increased trophic support and improved regenerative and repair properties. Therefore, Ti et al. demonstrated that LPS-prestimulated UCMSC-EVs upregulated the expression of anti-inflammatory cytokines and promoted M2 macrophage activation. The let-7b/TLR4 pathway was identified as a potential contributor to macrophage polarization and inflammatory ablation. While in an *in vivo* setting, LPS pre-EVs demonstrated significant alleviation of inflammation and enhanced healing of diabetic cutaneous wounds (Ti et al., 2015). Monsel and others examined the therapeutic effects of hBMSC-EV in acute lung injury (ALI) in mice. Compared to standard EVs, TLR3-primed EVs elevated the expression of COX2 and IL-10, and decreased expression of TNF-α. The primed EVs also increased the phagocytic index of monocytes and further reduced the BAL fluid bacterial cfu count (Monsel et al., 2015). TLR4-Primed h-MSC (origin not mentioned) EVs exhibited a greater efficacy in counteracting the effects of LPS on microglia compared to EVs derived from MSCs without stimulation. Furthermore, TLR4-primed EVs exhibit greater effectiveness in inhibiting microglia and astrocyte activation, reducing amyloid deposition, preventing demyelination, mitigating memory loss, and improving motor and anxiety-like behavioral dysfunction, which demonstrated the enhanced immunoregulatory potential against neuro-inflammation triggered by LPS (Markoutsa et al., 2022). EVs from MSCs retain antimicrobial capacity as well. Priming hBMSC-EVs with Poly(I: C) enhanced antimicrobial characteristics, which led to the upregulation of EV proteins, out of which 21 miRs have been identified as crucial components in host defense and innate immunity (Pierce and Kurata, 2021).

In conclusion, MSC-derived EVs hold great potential as a cell-free therapeutic approach for bone regeneration through osteo-immunologic modulation although they have not been extensively used. Their diverse cargo and regenerative properties mediated by TLRs make them valuable tools for disease treatment and tissue regeneration, offering new possibilities for precision medicine and personalized therapies.

## 5 Design of TLR signaling-modulating biomaterials for bone regeneration

Bone regeneration biomaterials are designed to promote the healing and regeneration of damaged or lost bone tissue (Zhao et al., 2022). Through their unique properties, these biomaterials aim to accelerate bone healing and restore normal bone structure and function. The biomaterials mimic the properties of the extracellular matrix, enhance cellular adhesion, and stimulate the recruitment and differentiation of bone-forming cells, of which MSCs play the central role and have been already applied in biomaterial to enhance bone regeneration (Chen et al., 2016). The application strategies of MSCs involve various approaches to harness the regenerative potential of MSCs for promoting bone healing and tissue regeneration. MSCs can be directly seeded onto scaffolds or biomaterials designed for bone regeneration. The MSCs adhere and proliferate on the scaffold surface, providing a source of osteogenic cells. They can also be delivered to the site of bone injury or defect through various methods, such as direct injection, scaffolds, hydrogels, or tissue engineering constructs. Moreover, MSC-EVs carry bioactive molecules and can modulate cellular processes involved in bone healing, including cell proliferation, differentiation, and angiogenesis (Liu et al., 2018; Negrescu and Cimpean, 2021; Zhong et al., 2022).

TLR pathways play a significant role in the application of MSCs as reviewed. In biomaterial-induced bone regeneration, TLRs promoted osteointegration as well. Apart from TLRs stimulating MSCs directly, bone-regeneration biomaterials activating or inhibiting TLR pathways in immune cells especially in macrophages largely enhanced MSC osteogenic differentiation. Chen et al. reported that incorporating Sr-, Mg-, and Si-containing bioactive ceramic coatings onto materials inhibited the inflammatory reaction via the inhibition of Wnt5a/Ca2+ and TLR pathways of macrophages, hence upregulated osteogenic differentiation of BMSCs (Wu et al., 2014). By incorporating Boron into calcium silicate coating(B-CS), Lu et al. found that the B-CS coating decreased M1 polarization and converted macrophages to the M2 phenotype thus promoting BMSC osteogenesis via restraining the TLR signaling pathway which was proved by results that expression of MyD88, Ticam1, Ticam2, and NF-κB genes in macrophages co-cultured with B-CS coating was significantly decreased (Lu et al., 2019). Fan et al. proved that by incorporating BMSC-EVs onto the TA-modified sulfonated-PEEK scaffold, the polarization of macrophages into M2 phenotype and subsequent osteogenesis were activated. After ingestion of EVs in macrophages, miR that negatively regulated the TLR-NF-κB pathway increased, especially miR199a, indicating that EVs-coated scaffold can regulate macrophage polarization by miRNAs that inhibit NF-κB pathway (Fan et al., 2021).

In contrast, research activating the TLR pathway for better osteointegration was also reported. In Su et al.‘s study, micron-sized single-layer graphene oxide (GO) was used to coat on porous titanium surface (Ti-GO). Under physiological conditions, macrophages on Ti-GO showed moderate activation of inflammatory responses through activating the TLR pathway, with increased osteoinductive cytokine production(Su et al., 2020). Shi et al. prepared Cu-containing mesoporous silica nanospheres (Cu-MSNs) using an *in situ* one-pot synthesis strategy. Cu-MSNs were phagocytized by macrophages, leading to the switch to M1 extreme and a proper immune micro-environment by initiating pro-inflammatory cytokines, which further induced osteogenic, and angiogenic factors and suppressed osteoclastogenic factors such as OPG. The immune micro-environment which was partly modulated by the TLR3/4 pathway due to the upregulation of MyD88 and Ticam1/2 led to robust osteogenic differentiation of BMSCs via the activation of the OSM pathway (Shi et al., 2016). Zymosan was grafted onto the surface of the Ti implant. The increased production of proliferation, proangiogenic, pro-osteogenic, and immuno-suppressive cytokines in macrophages cultured with Zym-Ti revealed the safety and efficiency of modified Ti. Further results validated that zymosan specifically activated TLR on macrophages and modulated these cells into the pro-regenerative phenotype. The rat femur condyle defect models indicated that the coatings increased the integration of the Ti column with bone tissue without causing abnormality to the host (Shi et al., 2018).

In summary, TLRs play a crucial role in the application strategies of MSCs used in bone regeneration biomaterials. TLR activation or inhibition on MSCs and other cells involved in osteointegration can enhance their immunomodulatory effects, promote osteogenic differentiation, and modulate the inflammatory response. Harnessing the potential of TLR signaling in MSC-based bone regeneration strategies holds promise for optimizing and improving bone healing processes.

## 6 Conclusion and perspective

The role of TLRs on MSCs in osteogenic differentiation, immunoregulatory potential, and therapeutic effects in bone tissue repair and other diseases is an active area of research, and the understanding of the specific mechanisms involved is still evolving. Studies have suggested that TLR signaling can influence the osteogenic potential of MSCs in several ways: 1) TLR signaling activation of MSCs can impact the expression of key genes involved in MSC osteogenic differentiation; 2) Inflammatory cytokines regulate bone formation and bone resorption following TLR stimulation of immune cells or MSCs; 3) The crosstalk between MSCs and immune cells following TLR stimulation is crucial for bone remodeling. TLRs can also indirectly influence MSC osteogenic effects by acting on other cells, such as immune cells, within the osteogenic microenvironment; 4) MSC-secreted EVs could influence or be influenced by TLR pathways to enhance the therapeutic effects. The interplay between TLRs and MSCs is a dynamic and multifaceted relationship that influences bone healing and tissue regeneration. Understanding and leveraging this interplay can contribute to the development of more effective strategies for enhancing bone healing and tissue regeneration.

The TLR pathway, as a complex signaling network, plays a significant role in the regulation of inflammation and MSC osteogenic differentiation. Based on the literature review, we conclude that controlling inflammation at an appropriate level can enhance the osteogenic potential of MSCs, and excessive inflammation may cause bone resorption. Given the diverse biological effects and cell-specific targeting of the TLR signaling pathway, future research should focus on appropriately activating inflammation or the TLR pathway to drive MSC osteogenesis through multiple influencing factors.
